# Human Cellular Models for the Investigation of Lung Inflammation and Mucus Production in Cystic Fibrosis

**DOI:** 10.1155/2018/3839803

**Published:** 2018-11-15

**Authors:** Stefano Castellani, Sante Di Gioia, Lorena di Toma, Massimo Conese

**Affiliations:** Laboratory of Regenerative and Experimental Medicine, Department of Medical and Surgical Sciences, University of Foggia, Foggia, Italy

## Abstract

Chronic inflammation, oxidative stress, mucus plugging, airway remodeling, and respiratory infections are the hallmarks of the cystic fibrosis (CF) lung disease. The airway epithelium is central in the innate immune responses to pathogens colonizing the airways, since it is involved in mucociliary clearance, senses the presence of pathogens, elicits an inflammatory response, orchestrates adaptive immunity, and activates mesenchymal cells. In this review, we focus on cellular models of the human CF airway epithelium that have been used for studying mucus production, inflammatory response, and airway remodeling, with particular reference to two- and three-dimensional cultures that better recapitulate the native airway epithelium. Cocultures of airway epithelial cells, macrophages, dendritic cells, and fibroblasts are instrumental in disease modeling, drug discovery, and identification of novel therapeutic targets. Nevertheless, they have to be implemented in the CF field yet. Finally, novel systems hijacking on tissue engineering, including three-dimensional cocultures, decellularized lungs, microfluidic devices, and lung organoids formed in bioreactors, will lead the generation of relevant human preclinical respiratory models a step forward.

## 1. Introduction

Cystic fibrosis (CF) is a recessive autosomal disease caused by mutations in the *CFTR* (cystic fibrosis transmembrane conductance regulator) gene located on the long arm of chromosome 7. Although CF is a multiorgan syndrome, lung disease represents the main cause of morbidity and mortality. More than 2000 mutations in the *CFTR* gene have been recorded (http://www.genet.sickkids.on.ca); however, the most common mutation associated with CF is a deletion of a phenylalanine in position 508 (F508delCFTR) which determines a misfolded protein that, although partially functional and sensible to cAMP/PKA-dependent regulation, is unable to reach the plasmatic membrane for its rapid degradation in the endoplasmic reticulum. The loss of a functional CFTR on the apical side of the respiratory epithelium causes an alteration of mucociliary clearance [1] with opportunistic pathogen infections [2] and chronic inflammation [3–5]. *Haemophilus influenzae* and *Staphylococcus aureus* are the primary microorganisms infecting the airways of infants and children with CF, followed by the *Pseudomonas aeruginosa* or *Burkholderia cepacia* complex during adulthood, although the CF infection is thought to be polymicrobial with viruses and fungi also involved [6].

**Figure 1:**
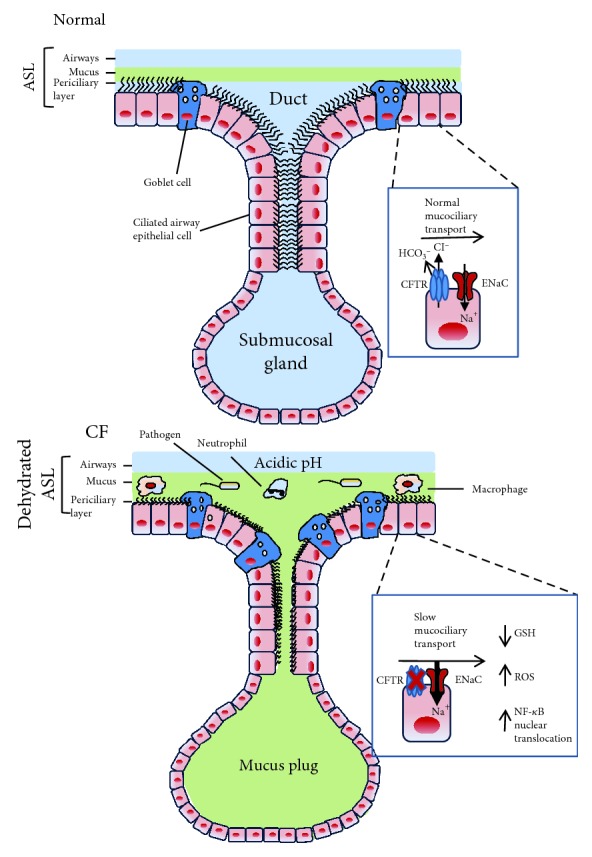
Pathophysiology of CF lung disease. (a) In the healthy state, the CFTR protein inhibits the epithelial sodium channel (ENaC), thereby regulating the absorption of sodium and water from the airway lumen, providing the adequate airway surface homeostasis with effective transport of mucus extruding from the airway surface goblet cells and submucosal glands. Physiological bicarbonate and pH regulation facilitates the formation of an airway surface liquid (ASL) that optimizes mucociliary clearance. Moreover, CFTR regulates transepithelial reduced glutathione (GSH) transport, maintaining the redox potential in the airways. (b) In CF, the absence of CFTR on the apical membrane leads to hyperactivity of ENaC, resulting in hyperabsorption of Na^+^ and water and consequently in reduction of the periciliary liquid (PCL) layer. Mucus transport slows down due to the incapacity of cilia beating with disruption of mucociliary clearance, contributing to mucus stasis also given by goblet cell hyperplasia and submucosal gland hypertrophy. Decreased bicarbonate transport contributes to an acidic pH. Moreover, lower levels of GSH contribute to increased concentration of reactive oxygen species (ROS). This oxidative stress leads to a heightened NF-*κ*B translocation in the nuclei of airway epithelial cells. These events contribute to a proinflammatory airway environment characterized by a massive neutrophil infiltration. Dysregulated macrophages contribute to the high inflammatory burden.

Mounting evidence has emerged on the role of CFTR as a protein with multiple functions, including the regulation of other channels. Within the airway epithelial cells, the CFTR protein exerts a tonic inhibition on the epithelial sodium channel (ENaC), thereby regulating the absorption of sodium and water from the airway lumen. In CF, the lack of CFTR on the apical membrane unchains ENaC that becomes hyperactive, ensuing hyperabsorption of Na^+^ and water from the periciliary fluid (PCL) that becomes thinner [7] (Figure 1). Subsequently, the mucus layer overlying PCL is not transported correctly due to the incapacity of cilia beating with disruption of mucociliary clearance. Primary cultures of airway epithelial cells have been instrumental in recognizing this pathomechanism [8]. Moreover, abnormalities of mucus and mucus-producing cells in CF have also been observed, although during the progression of lung diseases, including increased luminal mucus (with increased amounts of DNA derived from neutrophils), abnormal amounts of mucins (MUC5AC, MUC5B, and MUC2), goblet cell hyperplasia, and submucosal gland hypertrophy [9] (Figure 1). CF submucosal glands secrete strands and blobs of mucus that fail to detach from gland ducts, interfering with mucociliary transport [10].

Besides chloride, CFTR transports bicarbonate and thus, in CF, the loss of bicarbonate secretion determines an acidic airway surface liquid pH with subsequent impaired killing of pathogens and their removal from the airways [10] (Figure 1). Overall, it seems that multiple defects dependent on abnormal CFTR concur in failure to eradicate opportunistic pathogens from the CF airways.

Finally, it should be remembered that certain studies in cells and animal models have shown that CFTR has a role in transepithelial reduced glutathione (GSH) transport, thus maintaining the intracellular and redox potential in airways [11–14]. In CF airways, an increased concentration of reactive oxygen species (ROS) and lower levels of GSH have been reported [14] (Figure 1). This oxidative stress may lead to a heightened NF-*κ*B activation and translocation in the nuclei of airway epithelial cells, contributing to the hyperinflammatory response.

The primary scope of this review is to present and discuss the in vitro and ex vivo models of the airway epithelium obtained from human specimens; secondly, we will highlight the role of airway epithelial cells and their interplay with other immune cells in the generation of inflammation and mucus abnormalities in CF. Although animal models of CF are available, some of them do not present the hallmarks of the human lung disease [15–17]. CF mice do not exhibit the severe pathology characteristic of established human CF lung disease consisting of chronic respiratory infection, inflammation, mucus plugging, and progressive bronchiectasis [18]. The lack of these features can be traced back to different reasons, among which are alternative chloride channels that supply the deficiency of CFTR and anatomical differences since human airways have numerous submucosal glands throughout the trachea and bronchi, while murine airways only have a small proportion of these glands in the larynx and proximal trachea [19]. Other animal models have so far been established, including the rat [20], the ferret [21], and the pig [22, 23]. In this review, we will emphasize similarities and differences between human-derived cell models and animal findings in relation with inflammation as well as mucus production and lung disease.

## 2. The Inflammatory Response in CF Airways

The inflammatory response in CF lungs is characterized at the onset by the activation of innate immune cells, particularly airway epithelial cells (AECs) and macrophages, that recognize pathogens via pattern recognition receptors (PRRs), followed by the involvement of neutrophils [24]. The recruitment of neutrophils in the CF airways occurs through the release of chemoattractants, including IL-8, LTB_4_, C5a, C5a-des-Arg, high-mobility group box 1 (HMGB1), IL-17, proline-glycine-proline (PGP), N-acetyl PGP, and bacterial products such as N-formyl-methionyl-leucyl-phenylalanine (fMLP) [25]. We and others have analyzed cytokines, chemokines, and growth factors, implied in inflammation and remodeling of the CF airways, finding higher levels of these mediators in diverse specimens obtained from the sputum and bronchoalveolar lavage fluid (BALF) of CF patients (reviewed in [26, 27]). In the CF airways, neutrophils often undergo necrosis rather than clearance by normal apoptotic mechanisms. Dying neutrophils release large amounts of intracellular contents, including actin and long-stranded DNA, which contribute to the high viscosity of CF sputum [28, 29]. Moreover, neutrophils are disabled once they have entered the CF airways. The main culprit for this effect is represented by proteases secreted by neutrophils themselves. Neutrophil elastase (NE) has several potential roles in disabling neutrophils including cleavage of opsonophagocytosis proteins, such as iC3b, complement receptor 1 (CR1) and C5a receptor, the chemokine receptor CXR1, and TIM3 receptor leading to decreased galectin 9/TIM3 interactions [30]. Overall, the loss of these proteins is responsible for suboptimal local neutrophil priming and bacterial clearance.

The main protective functions of the airway epithelium against pathogens are to provide a barrier, represented by tight junction (TJ) complexes maintaining the structural integrity of the epithelial layer between the cells. AECs sense the presence of pathogens and orchestrate the following steps in the host response [24, 31]. Innate cell populations such as macrophages are constantly present in the airways and the alveoli; a whole population of dendritic cells (DCs) reside inside and underneath the airway epithelium [32, 33]. The role of macrophages and dendritic cells in the CF lung disease is being unrevealed in recent times, and it has been covered by excellent reviews [34, 35] which we refer to the reader.

## 3. Mucus Production and Airway Remodeling in CF Airways

Mucus is a mixture of water, glycoproteins, ions, and lipids that constitutes a physiological barrier that protects the apical surfaces of the respiratory, gastrointestinal, and reproductive epithelial tracts, whose components are secreted apically by epithelial and glandular cells. The main macromolecular constituents of mucus are large O-glycosylated proteins called mucins (MUC) which are encoded by *MUC* genes, with MUC5AC (secreted by goblet cells) and MUC5B (secreted by submucosal glands and goblet cells) being the predominant mucins in lung secretions [36]. CF patients overproduce airway mucins, reflecting goblet cell hyperplasia in the airway epithelium. Several possible mechanisms have been proposed to establish a correlation between CFTR deficiency and mucus obstruction in different organs, hypothesizing that epithelial CFTR could be involved directly in mucus production [37] or indirectly by contributing to the ionic drive needed for the physiological hydration of the mucus layer [38]. Interestingly, mucin secretion in primary CF AECs is normal and comparable to that of non-CF cells [39] and does not appear to be directly linked to lack/dysfunction of CFTR as demonstrated by experiments on non-CF cells treated with CFTR inhibitor Inh172 [39]. Moreover, the concept that several stimuli upregulate *MUC5AC* and *MUC5B* genes in lung epithelial cells with no evidence of a direct role of CFTR has been reinforced by different studies. In this sense, the mouse model overexpressing the *β*-subunit of ENaC in the lungs is a very good demonstration of this assumption. In this model, CFTR expression and function are not altered, but nevertheless *β*-ENaC mice exhibit hallmarks of CF lung disease including mucus hypersecretion, mucus obstruction in the conducting airways, mucociliary clearance impairment, goblet cell metaplasia, and neutrophilic airway inflammation [40–42]. Conversely, CFTR knockout ferrets and CF pigs (comprising CFTR^−/−^, CFTR^F508/F508del^, and CFTR^-/F508del^) demonstrate a severe lung phenotype at birth as concerning mucus plugging in the small and large airways [43–46], indicating that the lack of CFTR expression and/or function is fundamental for lung disease initiation and progression in these larger animal models, mimicking human disease.

Transcriptional upregulation of *MUC* genes is activated by a wide variety of stimuli present in the airways of CF patients, e.g., *P. aeruginosa* components (i.e., lipopolysaccharide (LPS) and flagellin) and proinflammatory cytokines. In particular, *P. aeruginosa* components, recognized by toll-like receptors (TLR), transcriptionally upregulate *MUC2* and *MUC5AC* expression via the canonical NF-*κ*B pathway. Overproduction of mucins in CF is also known to be induced by the epidermal growth factor receptor (EGFR) activated by transforming growth factor-*α* (TGF-*α*) that is strongly increased in the epithelium of patients with CF [47]. EGFR activates extracellular signal-related kinase (ERK) 1,2 cascade leading to the activation of c-Jun/c-Fos and SP1 transcription factors that can trigger *MUC5AC* and *MUC2* transcription.

Neutrophils, the main inflammatory cells in CF airways, secrete neutrophil elastase, which increases *MUC5AC* gene expression in A549 (type II penumocytes), differentiated human bronchial epithelial cells at air-liquid interface (ALI), and NCI-H292 lung mucoepidermoid cells [48, 49]. Finally, defensin human neutrophil peptide-1 (HNP-1), a neutrophil-derived antimicrobial peptide, also increases levels of *MUC5AC* in NCI-H292 cells and induces phosphorylation of ERK1/2 [50].

Data obtained from cell cultures have found that proteases, oxidants, pathogen virulence factors, and inflammatory mediators induce airway epithelial damage [51, 52] that progressively with time ensues in airway remodeling, consisting in basal cell hyperplasia, increase in epithelial height, squamous metaplasia, and cell shedding [53–58]. However, endobronchial biopsies provide the picture that airway remodeling starts early in the life of CF individuals [59]. Notably, some remodeling features seem to be somehow CFTR-specific, since delayed and abnormal epithelial regeneration, epithelial thickening, increased reticular basement membrane thickness, and mucus cell number appeared to be independent of infection and inflammation [60, 61].

The mechanism of airway remodeling in CF has not been revealed yet. Some studies have shown that active CFTR suppresses EGFR-related proinflammatory responses, including IL-8 secretion and mucus production [62–64]. The transmembrane protease ADAM17 (a disintegrin and metalloproteinase 17) sheds the soluble EGFR binding domain of transmembrane growth factors and cytokines in the extracellular milieu, allowing both autocrine signaling toward the epithelial cells and paracrine epithelial-mesenchymal *trans*-signaling [65]. This loop might be important in amplifying mucus production and fibrotic phenomena related to airway remodeling.

## 4. Models of Cell Cultures

**Figure 2:**
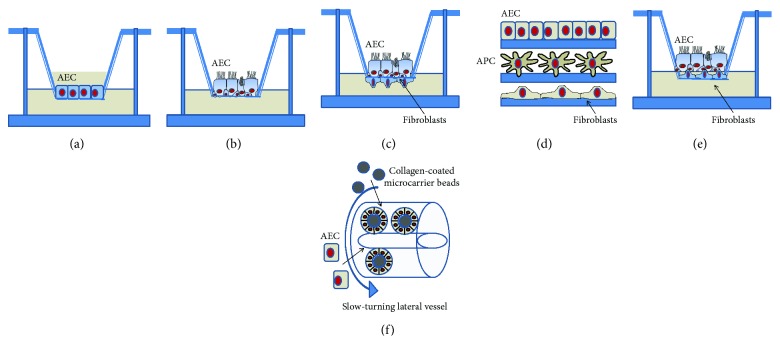
Models of AEC culture. 2D (a, b, c) and 3D (d, e, f) models are shown. AECs are traditionally cultured onto filter inserts submerged in media until they polarize (a) or differentiate under ALI condition (b). While secondary cell lines polarize but not differentiate at ALI (e.g., Calu-3), primary AECs differentiate into a pseudostratified epithelium presenting ciliated, basal, and mucus-producing goblet cells. These filter inserts can then be removed and upended to allow the attachment of accompanying cell types before the filters are placed back in the well (c). Fibroblasts are shown; however, they could be other cell types, such as DCs or macrophages. An immunocompetent 3D model of the human upper airway was developed using the epithelium, antigen-presenting cells (APC), such as DCs, and fibroblasts (d). Different cell types can individually grow on support and then layered one on top of the other or directly grow in this way. Another 3D model is based on the deposition of human pulmonary fibroblasts onto a collagen-type IV-coated insert, followed by the seeding of AECs and culturing them first in submerged conditions and then at ALI (e). RWV (rotating wall vessel) technology allows the growth of AECs on the surface of porous collagen-coated microcarrier beads in suspension under low fluid shear and gentle mixed conditions inside cylindrical bioreactors, termed slow-turning lateral vessels (STLV) or high aspect ratio vessels (HARV) (f).

In the following sections, we have discussed the models of AECs used to study and evaluate the inflammatory response and mucus production in their various aspects. Models include simple culture on plastic dishes or wells, culture on permeable inserts that allow cell polarization, cocultures of airway epithelial cells with immune cells, and also three-dimensional (3D) epithelial-mesenchymal cocultures (Figure 2). Cultures on inserts can be performed either with the submerged epithelium or at ALI. Epithelial cell cultures can be derived from secondary immortalized cell lines, primary cells, and stem/progenitor cells. Secondary cell lines that are most used in the CF field are listed in Table 1.

### 4.1. Cultures on Plastic Supports

Cells grown on plastic dishes have been used initially in the characterization of inflammatory properties of AECs [66]. Typically, cells are cultured on coatings made by extracellular matrix proteins, such as fibronectin and collagen as well as bovine serum albumin, and the culture medium is supplemented with bovine serum.

Using this type of culture, various results have been obtained as concerning the expression of PRRs, among which the most studied are TLRs. Greene et al. [67] found that that TLR1–5 and TLR9 are expressed on the surface of CF (CFTE29o– and CFBE41o–) and non-CF (16HBE14o–) tracheal and bronchial epithelial cell lines. On the other hand, it has been reported that TLR4 is not surface expressed on BEAS-2B tracheobronchial and A549 cells [68], suggesting that culture on coated plates brings to heterogeneous results, likely because it is far from the anatomical reality. This is paralleled by the mucus production. Indeed, while the primary airway epithelium grown at ALI has a mucus layer, cell lines, such as A549 and BEAS-2B, when grown on plastic as two-dimensional (2D) monolayers, show a very low or absent mucus content [69].

Submerged cultures have been also used to study the relevance of EGFR and ADAM17 activity on the inflammatory response in both NCI-H292 cell line and wild-type primary AECs [63]. The secretion of IL-8 and IL-1*α* cytokines was increased by the treatment with the CFTR inhibitor Inh172 via a mechanism dependent on EGFR and ADAM17. These results were confirmed when the effects of CFTR on constitutive IL-8 production in airway epithelial (IB3) cells containing mutant CFTR and in isogenic (C38) cells complemented with wild-type CFTR were studied. However, it is known that Inh172 has off-target effects [70] and increases IL-8 secretion in the bronchial CF line CFBE41o– in ALI culture [71], suggesting at least in part a CFTR-independent component in the response to Inh172. Moreover, a comparison of CFTR-deficient IB3 and its wild-type corrected variant C38 in signals downstream to CFTR (e.g., IL-8 secretion) is problematic since these cell lines are subject to clonal variation and have been cultured separately for a long time.

Changes in the transcriptional response of CF compared to non-CF epithelial cells after *P. aeruginosa* infection have been studied in submerged cultures with variable results. The main reason is that different cell sources have been investigated. In one study, infected CF cells (IB3 cell line) overexpressed proinflammatory mediators such as cytokines, chemokines, and growth factors but underexpressed protease inhibitors, compared to control cells (S9 cell line, that is, IB3 cells transduced with a CFTR-expressing adenovirus) [72]. In a more recent study with primary bronchial epithelial cells [73], genes belonging to the same biological families were described although not exactly matching those previously identified. In contrast, another study produced contradictory results to those cited, with higher levels of IL-8, IL-6, and ICAM-1 expression in control cells (CFT1-LCFSN) compared to CF cells (CFT1-ΔF508) after infection [74].

NuLi-1 cells were isolated from a non-CF patient expressing wild-type (wt) CFTR, whereas CuFi-1 cells are a CF cell line homozygous for F508 mutation. NuLi-1 cells, when exposed to the PAO1 strain of *P. aeruginosa*, show a higher proinflammatory profile than CuFi-1 cells, as shown by the hypersecretion of IL-8 and higher expression of ICAM-1, an adhesion molecule involved in neutrophil epithelial transmigration [75].

Overall, different experimental protocols and cell sources, as well as the lack of a well-differentiated epithelium, preclude any general consideration on the relevance of these data in relation to inflammation, mucus production, and response to the CF pathogens.

### 4.2. Cells Grown on Semipermeable Filters

In order to obtain a differentiated airway epithelium mimicking in vivo situation, ALI culture can be employed [76]. This method is widely used for both immortalized and primary cells, although cells grown at the liquid-liquid interface can deliver some interesting results (Figure 2(a)).

Cell lines form a polarized monolayer but do not differentiate; however, they show expected trans-epithelial activity in ALI condition that mimics the behavior of airway epithelial cells *in vivo* [77–80] and can allow studying some features of host-pathogen interaction [69]. Primary hAECs originate from nasal turbinates, nasal polyps, trachea, and bronchi, and established protocols allow them to be cultured onto semipermeable supports [81]. Using these protocols, primary bronchial epithelial cells in the bronchial epithelial growth medium (BEGM) are seeded onto the apical chamber of either 6.5, 12, or 24 mm Transwell inserts coated with type IV collagen and with media in the basal chamber. Upon reaching cell confluency, the apical medium is removed and the basal medium switched to air-liquid interface-specific media, essentially a 50 : 50 mixture of BEGM with DMEM-H [82]. Cells are then cultured for 2–4 weeks to allow proper differentiation and polarization. Primary AECs on ALI conditions are polarized and pseudostratified and differentiate in a mixed population (including ciliated, basal, and goblet cells) expressing beating cilia, producing mucus, and forming tight junctions, whose organization could be measured by transepithelial resistance (TER) [83] (Figure 2(b)). CF and non-CF cultures, either immortalized or primary, can be then mounted in Ussing chambers and studied for cAMP-mediated Cl^−^ conductance and ENaC- and Ca^2+^-activated Cl^−^ channel. Thus, CF epithelia show absence of cAMP-mediated Cl^−^ currents, a greater percentage inhibition of the basal current by amiloride (reflecting hyperactivity of ENaC), and an elevated function of the Ca^2+^-activated Cl^−^ conductance as measured by the response to uridine triphosphate [82]. Epithelia obtained from ALI cultures have a different cell composition when compared with epithelial cells brushed from the large airways: ALI cultures of large airway epithelial cells derived from a healthy nonsmoker had higher percentages of basal cells, whereas brushed cells collected in vivo from healthy nonsmokers presented higher percentages of ciliated cells [84]. Despite this, ALI cultures provide a good surrogate of the *in vivo* airway epithelium, since a similar transcriptional profile has been demonstrated by comparing the genome-wide expression of tracheal and bronchial epithelia *in vivo* with that of primary cultures of human AECs grown at ALI conditions [84]. Another study [85] confirmed these results, with some differences in transcriptome reflecting the differential composition of cell types.

The ALI culture has been fundamental to understanding molecular pathways leading to CF inflammation pathomechanisms in CF and response to pathogens since they recapitulate epithelial polarity of the *in vivo* tissue in both disease and health condition. The patterns of TLR distribution in the CF and control cell lines are similar when transformed cell lines (16HBE14o–) and cells in primary culture were analyzed in ALI conditions [86]. Overall, these findings, together with those obtained in cell culture on dishes, indicate that TLR expression is not dependent on CFTR status and function [87].

In order to investigate the role of oxidative stress in CF, bronchial cell lines were compared with primary airway epithelial cells grown at ALI [88]. Several transcripts already reported to be upregulated in CF pathophysiology (IL-6, IL-8, IL-1B, and IL-1A) were found to be significantly higher in CuFi-1 cells as compared with NuLi-1 cells. Also related to inflammatory processes, genes of the alarmin family were shown to be upregulated in CF cells, such as IL-33, lactotransferrin, heat shock protein 70, defensin beta 4, and calcium-chelating S100 family proteins (also called calgranulins S100A8-A9-A12). Upon oxidative stress, as induced by DMNQ (2,3-dimethoxy-1,4-naphthoquinine), the gene expression profile of CuFi-1 cells exhibited a modulation of genes involved in survival, which resulted in a net antiapoptotic effect. These results were confirmed in another pair of immortalized cell lines (CFBE41o– and CFBE41o– transduced with wt-CFTR [corrCFBE41o–]), as well as when comparing CF vs. non-CF primary culture of bronchial epithelial cells.

ALI cultures obtained from AECs originating from different airway regions have identified differences related to physiology and inflammation [89]. CF nasal cell, obtained from brushing of CF patients, and grown at ALI revealed the presence of ciliated, mucus-secreting and basal cells, and typical TJs. CFTR protein expression was observed in CF (F508del/F508del) and healthy cultures and ciliary beating frequency was similar to other cell types grown in similar conditions. In terms of cytokine secretion, baseline IL-8 (~800 pg/mL) and IL-6 (~100 pg/mL) release was similar in control and CF ALI cultures. Becker et al. [90] had previously shown that ALI cultures obtained from lower airways (trachea and bronchi) behaved similarly to nasal cells: unstimulated non-CF and CF cultures produced similar amounts of IL-8 although at higher levels than nasal cells (2.3 ± 0.5 and 1.6 ± 0.4 ng/mL, respectively), indicating that upper and lower airways are not fully superimposable as concerning cytokine production. Since airway epithelial nasal cultures are increasingly used as a surrogate to bronchial cells [91–93], this limitation should be taken into account for a correct interpretation of the results.

**Figure 3:**
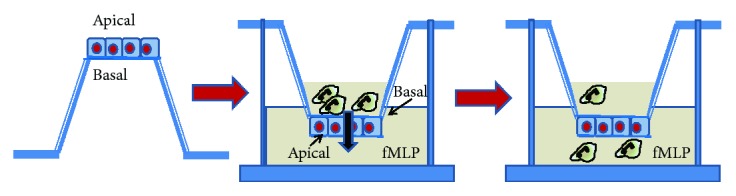
Neutrophil transmigration in AEC culture. AECs are seeded on the opposite side of filter inserts and allowed to attach, then the insert is inverted and can be cultured either submerged or at ALI (not shown). Neutrophils are added on the top chamber representing the basolateral surface of AECs and induced to migrate to the lower chamber against a chemoattractive agent (e.g., fMLP). After 3–4 hours, neutrophils are recovered from the lower chamber and percentage of migrated cells over the total is calculated.

More recently, an advanced coculture model with primary human airway basal cells has been developed to explore neutrophil transepithelial migration. In order to obtain a directionally relevant neutrophil transepithelial migration, a conventional ALI culture was performed by seeding the human AECs on the underside of the Transwell [94]. This primary coculture model recapitulates, in a more physiological fashion, key molecular mechanisms that regulate bacteria-induced neutrophil transmigration previously characterized using an immortalized human AEC line model (Figure 3). In a previous work, we have provided evidence, in a submerged model, that CF airway epithelial cell monolayers (CFBE41o–) have a leaky barrier function, allowing a pathophysiologically relevant higher neutrophil transmigration (basal to apical) [95]. The leakiness of the epithelial monolayer was partially reduced by CFTR relocation to the apical plasma membrane by overexpressing Na^+^/H^+^ exchanger regulatory factor 1 (NHERF1), a protein involved in PKA-dependent activation of CFTR and part of a multiprotein complex that via actin cytoskeleton reorganization stabilizes F508del CFTR on the apical membrane tethering it to the cytoskeleton.

Forrest et al. [96] used 200 *μ*m thick inert 3D scaffolds with >90% porosity (pore sizes of 36–40 *μ*m, with interconnects of 12–14 *μ*m) and coated with collagen on top of which H441 cells were initially grown at ALI. After intact epithelial layers had formed, neutrophils were loaded onto the basal compartment of the scaffold (situated upside) and allowed to migrate through the collagen and epithelial layers into the apical compartment (situated downside) and bathed with either a control medium with chemoattractant or an airway supernatant obtained from CF sputum. Transmigrated neutrophils acquired phenotypes similar to those found in airways of the patients, including cell surface loss of CD16 (Fc*γ*RIIIB, the low affinity receptor for IgG) and increased expression of CD63 (used as a surrogate for exocytic release of elastase). These changes did not appear if the neutrophils were cultured directly in the medium containing lung fluid from CF patients; nor did they appear if transepithelial migration was toward the leukotriene LTB4.

Recently, it has been shown that CF bronchial epithelial cells in ALI display a defect in acid ceramidase (Ac) as compared to cells obtained from non-CF individuals [97]. The reduction in Ac caused an accumulation of ceramide on the apical surface of AECs associated with increased *β*1 integrin expression and low sphingosine levels. The combination of low sphingosine concentrations and increased release of dead cells or DNA from the epithelial cell layer into the lumen causes the high susceptibility to *P. aeruginosa* infection. Increased ceramide levels also trigger the activation of CD95 and thereby mediate increased death of epithelial cells [98, 99].

ALI cultures of primary nasal cells have been investigated for epithelium remodeling [100]. These studies elucidated that even in the absence of exogenous inflammation, the regenerated CF epithelium is remodeled, exhibiting basal cell hyperplasia and a delayed ciliated cell differentiation as compared to the non-CF counterpart. The diminished number of ciliated cells would worsen the mucociliary clearance impairment leading to further infection and inflammation. Mucus cell hyperplasia appeared only if the CF epithelium was challenged with cytokines found elevated in the CF sputum (TNF-*α*, IL-1*β*, and IFN-*γ*), suggesting a vicious cycle integrating inflammation and remodeling. Nevertheless, the role of inflammatory stimuli on ion transport in airway epithelial cells and goblet cell maturation has been highlighted [101]. For example, we have shown that IFN-*γ* (a Th1 cytokine) decreased CFTR-dependent Cl^−^ secretion by more than 60% at 48 hours of treatment in polarized bronchial epithelia (grown at ALI) [102]. Conversely, Th2 cytokines, such as IL-4 and IL-13, have been shown to upregulate Ca2^+^-dependent Cl^−^ secretion in bronchial epithelial cells at ALI [103] and at the liquid-liquid interface [104]. Scudieri et al. [105] have subsequently shown that primary bronchial epithelial cells grown at ALI when treated for 24 h with IL-4 displayed a marked increase in TMEM16A protein expression, an important component of Ca2^+^-dependent Cl^−^ secretion. In parallel, IL-4 caused a decrease in the percentage of ciliated cells, whereas it increased the percentage of MUC5AC-positive cells, in agreement with the induction of mucus metaplasia by Th2 cytokines. The MUC5AC-expressing cells were also strongly positive for TMEM16A, representing a subset of the bronchial epithelial cells. Future studies should investigate whether TMEM16A upregulation also occurs in CF patients, as a consequence of bacterial infection and inflammation, or is a phenomenon specifically linked to the Th2 signaling cascade, as it has been suggested by other studies in CF patients [106].

The increased expression of transglutaminase 2 (TG2) has been related to epithelial-mesenchymal transition (EMT) in IB3 cells in submerged culture and also in more physiologically related conditions using ALI as well as in primary bronchial epithelial cells [107]. In both submerged and ALI culture conditions, TG2 activity inhibition in IB3 cells either with TG2 inhibitors or with specific knockdown of TG2 expression led to a significant reduction in fibronectin, N-cadherin, the transcriptional repressor Slug, and TGF*β*1 expression and in TGF*β*1 levels leading to a reversal of EMT. Similarly, when TG2 was overexpressed in primary bronchial epithelial cells grown under ALI conditions, an increase in expression of EMT markers could be observed at both gene and protein levels. These data indicate that TG2 would be implicated in increased matrix deposition leading to fibrosis in CF patients. It can be hypothesized that TGF*β*1, through its activation by TG2, is the driver of EMT progression in CF airway bronchial epithelial cells; however, it should be tested in primary CF AECs and *in vivo* in animal models.

The role of CFTR in the EGFR/ADAM17 axis has been investigated with an immortalized CF cell model (iCFTR CFBE) that allows inducible expression of CFTR in ALI culture [71], avoiding invalid comparisons of genetically distinct populations of immortalized cells. In ALI cultures, where they assume a low cuboidal morphology, not-induced iCFTR CFBE cells showed an enhancement of ADAM17-dependent shedding of amphiregulin, which, in turn, regulates EGFR activity, as compared with CFTR-expressing cells [108]. Importantly, these results were replicated in primary bronchial epithelial cells in ALI culture as well and allowed understanding that extracellular oxidative stress, likely related to deficiency in GSH transport, enhances the basal activity of the EGFR/ADAM17 axis through the ADAM17-dependent shedding of EGFR agonists causing enhanced EGFR activity [109].

Animal models can be instrumental in deciphering the initial mechanisms originating infection and inflammation in the CF airways and complement findings in cell cultures. Since both mouse and rats with CF do not display any spontaneous infection and inflammatory responses [15], the most attention in this field has been paid to larger animals, such as ferrets and pigs, that have similar lung architecture and physiology to human airways. Interestingly, proteomic analysis of BALF revealed that CF ferrets at birth show significant differences as compared with non-CF newborns in liver X receptor/retinoid X receptor (LXR/RXR), eukaryotic initiation factor 2 (eIF2), and mammalian target of rapamycin (mTOR) signaling pathways [110], all regulating inflammatory responses and interacting with the NF-*κ*B pathway. NF-*κ*B in turn regulates the expression of CF proinflammatory cytokines, such as IL-8, TNF-*α*, and IL-1*β* [86, 111, 112]. Notably, the concentrations of IL-1*β* and nitric oxide (NO) detected in newborn CF BALF were significantly lower than those in non-CF BALF, whereas those of IL-8 and TNF-*α* were significantly elevated. Some of these observations are in line with what has been seen in CF cells *in vitro* and in CF patients *in vivo*. NF-*κ*B and other signaling pathways have been shown to be hyperactivated in CF airway cells and are thought to contribute to their abnormal cytokine responses (including IL-8 and TNF-*α*) to bacterial and LPS challenge [113]. Some of the inflammatory markers elevated in newborn CF ferret BALF are also elevated in infants with CF [114, 115]. Moreover, the finding that NO is diminished in newborn CF ferret BALF mirrors observations in human patients [116]. These results are in stark contrast to what is known about the CF pig, which lacks lung inflammation at birth [22, 44]. BALF profiles indicate no difference in neutrophil infiltration and IL-8 concentration between newborn CF and non-CF pigs [22]. However, over time inflammation ensues, ranging from mild to severe leukocytic infiltration and heterogeneous airway remodeling, with evidence of goblet cell hyperplasia, airway wall thickening, and rarely distended submucosal glands [44]. This timing difference may be due to gross anatomical differences between ferrets and pigs and thus different CFTR-dependent innate immune responses. Moreover, the CF ferrets and pigs must still be interrogated in the inflammatory pathways that have emerged from human epithelial cell cultures. A vis-à-vis study of lung inflammation in newborn CF pigs in vivo and ex vivo showed that CF pig airway epithelia exhibited blunted early inflammatory responses to a *S. aureus* challenge [117]. In particular, whereas CF and non-CF pigs present similar tissue and BALF profiles in response to a 4-hour exposure to *S. aureus*, they show differing transcriptional profiles in their airway tissues, with diminished expression in genes encoding host defense response and increased gene expression in apoptotic response. Similar findings were reported when primary tracheal epithelial cells from CF and non-CF pigs at ALI were compared. More recently, it has been reported that monocyte-derived macrophages obtained from newborn CF pigs released more IL-8 and TNF-*α* in response to LPS [118], consistent with hyperresponsiveness to a TLR4 ligand [119]. These data are also consistent with an exuberant production of cytokines by CF murine macrophages in response to LPS [120].

### 4.3. Cocultures

An *in vitro* alternative to monoculture is coculture of different cell types including most commonly epithelial cells. This approach is being used to reduce the gap between simplistic single lineage *in vitro* models and the complex biological processes involving cytokines, growth factors, and transcriptional regulators that occur *in vivo* and has been employed to study the effects of neutrophils, eosinophils, monocytes, and lymphocytes on epithelial cell function. Cocultures of respiratory epithelial cells and immune cells are useful in mimicking the airway microenvironment in response to antigens or in developing competent uptake models for drug delivery [121].

AECs secrete a variety of proinflammatory cytokines and chemokines, which are all important for the cross-talk with antigen-presenting cells and epithelium in the immune responses [31, 122–125]. Cocultures can be done with either macrophages or dendritic cells, in order to investigate their response to particles, allergens, pathogens, and their subsequent effects on T cells [126–130] (Figure 2(c)). Triple cocultures with AECs, DCs, and macrophages or fibroblasts have been set up to study particle uptake [131], xenobiotic toxicity [132], defense against pathogens [128], and regulation of inflammatory response [133]. In preliminary studies, it has to be found which culture medium is appropriate for growth and acquirement of functional properties of the different cell types [134].

A coculture model of alveolar epithelial cells with monocyte-derived macrophages and dendritic cells has been used to model alveolar epithelial barriers to microorganisms [132].

Epithelial cell interaction with fibroblasts has been reported to modulate cell behavior, proliferation, and differentiation of the epithelium and vice versa [135]. For example, it is known that wound repair is dependent on the interaction of AEC actions with the extracellular matrix and on the cytokine milieu, established by themselves, but also bronchial wall fibroblasts, which secrete cytokines and modulate epithelial cell function [136] and ciliogenesis [137].

As concerning CF, it has been established that fibroblasts obtained from CF mice display an altered phenotype with increased proliferation and myofibroblast differentiation, higher sensitivity to growth factors, and overresponses of proinflammatory and fibrotic mediators [138]. However, no extensive studies on coculture of AECs and fibroblasts have been done so far.

An early study established long-term cultures (up to 10 months) of well-differentiated respiratory epithelia from CF nasal polyps, where postmitotic fibroblasts were generated by mitomycin C treatment and were then used as feeder layers seeded on the undersurface of Transwell inserts [139]. AECs were first grown submerged and then at ALI. Interestingly, CF cultures failed to show increased production of mucins and IL-8, but exhibited enhanced absorption of airway surface fluid. If this coculture system is advantageous in terms of expansion and CF AECs, it should be kept in mind that mitomycin C increases IL-8 secretion in corneal fibroblasts for example [140]. This effect may hamper the study of the inflammatory response under these experimental conditions and underline the importance for the use of actively proliferating fibroblasts in the investigation of the true proinflammatory responses by these cells.

In one PhD thesis work [141], human pulmonary fibroblasts (HPF) were seeded first onto the collagen IV-coated Transwells and incubated for four days before the epithelial cells (either C38, IB3-1, or Calu-3) were seeded on top. Further incubation under submerged conditions was allowed for the epithelial cells to adhere and proliferate before establishing ALI (Figure 1). Interestingly, all the coculture models were observed to able to establish a tight barrier and secrete MUC5AC into the apical secretions of each model over a period of at least 14 days. Monocultures and cocultures were challenged with live or heat-inactivated bacteria (*S. aureus*, *P. aeruginosa*, and *B. cepacia*) or with LPS and studied in terms of cell viability, TER, and IL-8 secretion. HPF clearly responded to bacterial challenges and could therefore play an important role in the pathogenesis of CF airway disease. Furthermore, this study showed evidence of a hyperinflammatory response in the CF model; thus, IB3-1 monocultures responded to challenge with significantly more IL-8 secretion compared to C38, which was also mostly the case in IB3-1-HPF cocultures. As to the epithelial integrity, when cultures were affected by live bacterial exposure, then mono- or cocultures of IB3-1 were usually more susceptible.

Direct cocultures of CFBE41o– cells with mesenchymal stem cells obtained from the amniotic membrane (hAMSCs) allowed us to investigate the functionality of the CFTR channel and the tightness of the epithelial barrier, which are altered in CF cell monolayers [95]. In these cocultures, at the mesenchymal-to-epithelial ratio of 5 : 1, we observed a recovery of the CFTR function as a chloride channel, a reduction of the fluid hyperabsorption from the apical surface, and an increased barrier function [142]. We have recently demonstrated that most of these effects are mediated by the formation of gap junctions between epithelial and mesenchymal cells [143], although the action of paracrine factors cannot be excluded. This coculture model can be further exploited for mimicking what would occur in vivo, following MSC transplantation to the lung, in relation to CF respiratory infections and inflammation.

### 4.4. 3D Epithelial-Mesenchymal-Immune Cell Cocultures

Conventional 2D platforms fail to represent the cellular arrangement seen *in vivo* and, moreover, are static. The use of a 3D tissue equivalent is favorable over 2D cell culture providing a more *in vivo*-like morphology, function, and intercellular interactions enabling greater resemblance to physiological conditions [144, 145]. These models represent invaluable tools in the fields of lung biology, disease modeling, and drug discovery and delivery. For example, a 3D model, in which human bronchial epithelial cells were applied onto a 3D fibroblast-embedded mesenchymal collagen layer, was recently employed to study collagen gel contraction and gene expression related to fibrogenesis [146] (Figure 2(e)).

A triple coculture has been established by a human lung tissue model composed of MRC-5 fibroblasts and a stratified 16HBE14o– epithelial cell layer in combination with monocyte-derived dendritic cells (DC) [147]. First, MRC-5 fibroblasts were seeded on collagen type I, then DCs were seeded on top of the stroma/matrix in submersion; finally, 16HBE was seeded on top of immune cells in a submerged fashion and lastly was exposed to the air. In this organotypic lung model, DCs were found to be located underneath, within, and on top of the epithelium and were also regulated in their production of chemokines CCL17, CCL18, and CCL22.

Harrington et al. [148] have used electrospun fibers of poly(ethylene terephthalate) (PET) to create a 3D model of the airway epithelium, comprising epithelial cells (Calu-3), DCs, and fibroblasts, cultured at ALI (Figure 2(d)), showing that cocultures recovered their integrity earlier than single cultures upon challenge with allergens and that they were amenable to DC migration from the bottom layer to the uppermost epithelial layer. 3D coculture models of AECs, immune cells, and fibroblasts have not implemented in the CF field yet.

## 5. Future Directions in Tissue Engineering

Furthermore, complex 3D models are necessary in mimicking the complex microenvironment in the lung mucosa. Spheroids can be obtained from either nasal polyps or lung tissue biopsies and show ciliogenesis and mucus production [149]. The advantages of this system are the very easy production (just gentle agitation) and the long-term culture (6 months when derived from nasal polyps and 6 weeks when derived from lung tissue). Due to downsides, including transcytosis, basolateral infection, and cellular organization (no single layer of pseudostratified epithelium) [150], this model has not further been implemented.

Recent advances in bioengineering technology have enabled the generation of lung organoid models. Tan and colleagues [151] have combined adult human primary bronchial epithelial cells, lung fibroblasts, and lung microvascular endothelial cells in 3D culture conditions, by using Matrigel, to generate airway organoids that are amenable to ectopic transplantation.

AECs can be grown as 3D aggregates when first allowed to adhere to collagen-coated beads and then placed into the rotating wall vessel (RWV) bioreactor, a horizontally rotating vessel that is completely filled with culture medium, called slow turning lateral vessel (STLV) or high aspect ratio vessels (HARV) [144] (Figure 2(f)). A549 3D aggregates showed improved characteristics as compared with monolayer cultures: formed a tighter epithelium, produced both MUC1 and MUC5AC on the apical surface of the 3D aggregates, indicating proper localization, which were more resistant to *P. aeruginosa* colonization and infection, and showed greater fold induction of IL-12, TNF-*α*, IL-10, and IL-6.

In a more advanced organotypic model, Crabbé et al. [152] added monocytes to the A549 3D aggregates and cocultured them in the STLV. They showed that monocytes differentiated to functional macrophage-like cells and localized on the alveolar surface with a macrophage-to-epithelial cell ratio relevant to an *in vivo* situation. Upon challenge with a quorum-sensing signal involved in the pathogenicity of *P. aeruginosa* infection in the CF lungs, the viability of macrophage-like cells was increased in the 3D model, indicating that alveolar cells and macrophages could interact to simulate a lung immunocompetent mucosal barrier.

Others have generated lung organoids by the agglomeration of cell-coated alginate beads in a HARV bioreactor and addition of fetal lung fibroblasts, small AECs (isolated from bronchioles), and human umbilical vein endothelial cells [153]. After 7 days, most of the small AECs presented surfactant protein B and C (type II alveolar cells) and T1a (type I alveolar cells), while a minority (less than 20%) were goblet and club cells (formerly known as Clara cells). Although the engineered multicellular organoids showed striking similarity with the native human lung alveolar structures, they did not show spontaneous formation of self-organizing capillary networks, indicating that other cues for vessel formation have to be explored.

In alternative, whole lungs can be treated to remove all cells and cellular materials leaving an intact 3D scaffold comprised of innate extracellular matrix proteins in a biomimetically similar 3D architecture, a process called decellularization [154]. However, this procedure is hampered by a shortage of donated lungs and scaffold immunogenicity and cannot be used in high-throughput applications. Repopulation of decellularized lungs has been reported using a number of different cell types, including fetal lung cells, endothelial cells, embryonic stem cells (ESC), fibroblasts, induced pluripotent stem cells (iPSC), primary or immortalized airway and alveolar epithelial cells, and bone marrow- or adipose-derived mesenchymal stem cells (MSCs), although only partial recellularization of the alveoli and airways was achieved [154–156]. However, it may depend on the possibility to obtain a cell source with low senescence. In a recent investigation, LaRanger et al. [157] have used conditionally reprogrammed (CR) human bronchial epithelial cells (HBECs) and recellularization of a mouse lung in a bioreactor system to recapitulate the upper and lower airways within only 12 days. In parallel experiments in vitro under ALI conditions, CR cells achieved the differentiation only in an upper epithelium and after 35 days. What is interesting is that CR CF HBECs showed the same behavior, opening various scenarios in which CR CF HBECs are a valuable resource for studying genetic and correction approaches as well as pharmacological interventions.

More sophisticated models based on tissue engineering are being developed, although they are not applied to the CF field yet. Presently, these models are based on scaffold materials, cell sheet technology, or microfluidic-based devices (lung-on-a-chip) that provide the adhesion, proliferation, and migratory support to the AECs and recapitulate tissue- and organ-level physiology [158]. For example, Blume et al. [159] fabricated a multichamber cultured device that integrates standard permeable filter supports containing fully differentiated bronchial epithelial cells which are perfused by basal media simulating the circulation of fluids in the tissue. The epithelium was well-organized, as judged by the actin cytoskeleton and occludin staining, and responded earlier and at higher magnitude to pollen as compared to static culture conditions in terms of IL-8 secretion.

## 6. Conclusions

More than twenty years of research have established the main features of the inflammatory response, mucus production, and host-pathogen interaction in the CF field, particularly in the lung disease. Monocultures under submerged and at air-liquid interface have been instrumental in gaining this knowledge. Primary airway epithelial cells offer the possibility of studying pathomechanisms of inflammation and mucus production in a situation closer to that *in vivo* and therefore should be preferred to immortalized cell lines, whose advantages are being easily manipulable by genetic means and can be cultured for the long term. Novel, valuable, and functional cocultures were also developed, which represent a more complex model system, as these include macrophages, DCs, and fibroblasts. They can help to elucidate key cues in cell communication and behavior and offer novel opportunities in the discovery of novel treatments. CF research should refine these models and complement the results obtained thereof with those gained in animal models. Finally, we aim to refine the even more complex 3D models that will fulfill research areas that need improvement, such as the multicellular complexity (including immune cells and members of the indigenous microbiota), the incorporation of relevant ECM proteins, and the integration of physical forces to mimic multiorgan signaling and interactions that naturally occur within the context of the specific tissue microenvironment of interest.

## Figures and Tables

**Table 1:** Most used secondary airway epithelial cells of non-CF and CF origin.

Secondary cell line^a^	Type
*Non-CF*	
BEAS-2B	Tracheobronchial
16HBE14o–	Bronchial
H441	Derived from a lung adenocarcinoma
NCI-H292	Derived from a pulmonary mucoepidermoid carcinoma (squamous differentiation)
C38 (IB3 corrected with an adenovirus)	Bronchial
S9 (IB3 corrected with an adenovirus)	Bronchial
CFT1-LCFSN	Bronchial (derived from a F508del homozygous patient, with a third wild-type allele)
Calu-3	Derived from a lung adenocarcinoma
NuLi-1	Bronchial

*CF*	
CFTE29o–	Tracheal (F508del homozygous)
CFBE41o–	Bronchial (F508del homozygous)
IB3	Bronchial (F508del/W1282X)
CFT1-F508del	Bronchial (derived from a F508del homozygous patient, with a third F508del allele)
CuFi-1	Bronchial

^a^All are of human origin. For references, refer to the text.
